# Association between free-living sleep and memory and attention in healthy adolescents

**DOI:** 10.1038/s41598-020-73774-x

**Published:** 2020-10-09

**Authors:** Runa Stefansdottir, Hilde Gundersen, Vaka Rognvaldsdottir, Alexander S. Lundervold, Sunna Gestsdottir, Sigridur L. Gudmundsdottir, Kong Y. Chen, Robert J. Brychta, Erlingur Johannsson

**Affiliations:** 1 Centre for Sports and Health Sciences, University of Iceland, Stakkahlid, 105 Reykjavík, Iceland; 2 Department of Sport, Food and Natural Sciences, Western Norway University of Applied Sciences, Bergen, Norway; 3 Department of Computer Science, Electrical Engineering, and Mathematical Sciences, Western Norway University of Applied Sciences, Bergen, Norway; 4 Mohn Medical Imaging and Visualization Centre, Haukeland University Hospital, Bergen, Norway; 5 Diabetes, Endocrinology, and Obesity Branch, National Institute of Diabetes and Digestive and Kidney Diseases, Bethesda, MD USA

**Keywords:** Sleep, Sleep deprivation, Attention, Short-term memory

## Abstract

In laboratory studies, imposed sleep restriction consistently reduces cognitive performance. However, the association between objectively measured, free-living sleep and cognitive function has not been studied in older adolescents. To address this gap, we measured one week of sleep with a wrist-worn GT3X+ actigraph in 160 adolescents (96 girls, 17.7 ± 0.3 years) followed by assessment of working memory with an n-back task and visual attention with a Posner cue-target task. Over the week, participants spent 7.1 ± 0.8 h/night in bed and slept 6.2 ± 0.8 h/night with 88.5 ± 4.8% efficiency and considerable intra-participant night-to-night variation, with a standard deviation in sleep duration of 1.2 ± 0.7 h. Sleep measures the night before cognitive testing were similar to weekly averages. Time in bed the night before cognitive testing was negatively associated with response times during the most challenging memory task (3-back; *p* = 0.005). However, sleep measures the night before did not correlate with performance on the attention task and weekly sleep parameters were not associated with either cognitive task. Our data suggests shorter acute free-living sleep may negatively impact difficult memory tasks, however the relationship between free-living sleep and cognitive task performance in healthy adolescents is less clear than that of laboratory findings, perhaps due to high night-to-night sleep variation.

## Introduction

The National Sleep Foundation recommends that teenagers aged 14 to 17 sleep 8–10 h a night in order to maintain overall health and well-being^1^. Yet, the Centers for Disease Control has shown that less than 30% of US teens report achieving the recommend amount of sleep and over two thirds report sleeping 7 h or less^2^. Short sleep in adolescents has been associated with increased risk of a variety of negative health outcomes, from metabolic complications^3,4^ to mental health issues^5^. One of the more consistent findings is a link between shortened sleep and impaired cognitive function^6,7^. Randomised clinical studies of adolescents have demonstrated that even partial sleep deprivation, i.e. shorter than recommended sleep duration without recovery sleep^8^, for 1–7 nights can deleteriously effect a wide range of cognitive functions including alertness^9^, visual attention^10^, cognitive processing speed^11^, and memory^12^. In some cases, even two nights of recovery sleep, analogous to the weekend “catch-up sleep” common amongst this age group, is not sufficient to completely restore cognitive performance^10,13^. The results of these clinical studies are also supported by large cross-sectional studies that find sleep quantity and quality can negatively affect academic performance amongst teenagers^14,15^. Poor sleep quality is also associated with lower performance on tasks of working memory^15^ and executive functioning^16^. Further, adequate sleep may be important for continued brain development during adolescence^17^, especially in frontal and parietal regions that underlie cognitive domains such as attention and memory^18^.

Evidence that short and disrupted sleep can deleteriously affect cognitive function is largely derived from controlled studies of sleep restriction. Most previous studies of the relationship between free-living sleep and cognitive function that did not include sleep interventions have relied on self-reported sleep measures^17,19^ or examined younger students^15,20,21^ or small (< 20 participants), selective samples^22^ using actigraphy. Thus, there is limited information about how free-living sleep patterns associate with cognitive function in non-clinical adolescent populations. After identifying widespread actigraphy-measured sleep curtailment in a longitudinal cohort of older Icelandic adolescents^23^, we included a cognitive assessment during a subsequent round of data collection to determine whether associations between sleep restriction and cognition identified in laboratory studies were also present in a free-living setting. Thus, the aim of the current study was to measure one week of free-living sleep using wrist actigraphy followed by testing of short-term working memory and visual attention in older adolescents. We tested whether the cognitive function of healthy adolescents was associated with free-living sleep duration and quality measured acutely, the night prior to testing, or cumulatively over an entire week. We hypothesized that shorter and more disrupted free-living sleep would be negatively associated with performance on tasks of short-term memory and visual attention.

## Results

### Participants

We conducted an exploratory analysis of the association between sleep and cognitive function, a secondary outcome in a longitudinal study of health and fitness from childhood through adolescence in Iceland^23,24^. The analysis included 160 participants that completed the cognitive tasks and a questionnaire which included self-reported videogame use and presence or absence of a clinical diagnosis for attention-deficit hyperactivity disorder (ADHD), met the criteria for a valid week of wrist actigraphy-measured sleep (≥ 3 weeknights and ≥ 1 weekend nights)^23^, and had valid sleep measures the night prior to cognitive testing. Participant characteristics are summarized in Table 1.

**Table 1:** Characteristics for participants with valid weekly sleep and valid sleep measured the night prior to cognitive testing.

Characteristics	Mean ± standard deviation or N (%)
N (% female)	160 (60.0%)
Age (years)	17.7 ± 0.3
Weight (kg)	68.7 ± 13.4
Height (cm)	173.9 ± 9.1
Body mass index (kg/m^2^)	22.7 ± 3.9
Videogame use (h/day)	0.8 ± 1.1
Clinical diagnosis of ADHD (N, %)	7 (4.3%)

*ADHD* attention deficit hyperactivity disorder.

### Sleep measures

A summary of all sleep parameters measured by wrist actigraphy is shown in Table 2. Average weekly rest duration (the time between bedtime and rise time) and sleep duration (time spent asleep) were 7.1 ± 0.8 h/night and 6.2 ± 0.8 h/night, respectively. This suggests that, on average, most participants did not spend the recommend 8–10 h/night in bed. Intra-individual nightly variation (i.e. standard deviation over all valid nights^23,25^) in sleep (1.2 ± 0.7 h) and rest durations (1.4 ± 0.8 h) over the week was also quite high. Measures of sleep quality included sleep efficiency (i.e. the ratio of sleep duration to rest duration multiplied by 100) which was 88.5 ± 4.8%, and minutes of wakefulness after sleep onset (WASO), which was 51.9 ± 20.5 min/night over the week. The average weekly mid-sleep time, a marker of sleep timing, was 04:48 ± 1.0 h. With the exception of an earlier mid-sleep time (04:19 ± 1.0 h, *p* < 0.001), sleep parameters on the night prior to cognitive testing were not significantly different than the weekly averages. The later average weekly mid-sleep time was likely caused by including weekend nights into the average, whereas all cognitive testing occurred after a school night. As we have demonstrated previously, students in this cohort tend shift toward later sleep schedules on the weekend^25^.

**Table 2:** Sleep measures for participants with valid weekly sleep and valid sleep measured the night prior to cognitive testing.

	Sleep the night prior to cognitive testing	Weekly sleep	*p* value
Mid-sleep time (clock time ± h)	04:19 ± 1.2	04:48 ± 1.0	** < 0.001**
Total rest time (h/night)	7.0 ± 1.4	7.1 ± 0.8	0.15
Total sleep time (h/night)	6.1 ± 1.4	6.2 ± 0.8	0.11
WASO (min/night)	51.4 ± 28.5	51.9 ± 20.5	0.71
Sleep efficiency (%)^a^	88.4 ± 7.1	88.5 ± 4.8	0.63
Total rest time variability (h)^a^		1.4 ± 0.8	
Total sleep time variability (h)^a^		1.2 ± 0.7	

Results presented as mean ± standard deviation, unless otherwise noted. *p* values are the result of paired t-tests comparing sleep the night prior to cognitive testing to weekly sleep. Boldface type indicates significant differences.

*WASO* wake after sleep onset.

^a^Results presented as median ± interquartile range due to skewed distributions.

### Cognitive measures

#### Short-term memory task

Short-term working memory was assessed using a numeric version of the n-back task. Positive, single-digit integers (i.e. 1–9) appeared one at a time in the centre of the screen in a fixed sequence. Participants indicated whether the current digit was the same as the one presented n positions back in the sequence, where n varied from 1 to 3, with higher numbers representing greater working memory load.

Performance on the N-back task was quantified using response time, or the time between stimulus appearance and participant response, and response accuracy, defined as the proportion of correct responses (i.e. the sum of correct button presses and correct rejections divided by total stimuli), as shown in Table 3. Response time and accuracy varied predictably across cognitive load: response time gradually increased while accuracy decreased in a dose–response manner from the 1-back to the 2-back and 3-back conditions (all *p* < 0.05).

**Table 3:** Cognitive measures for participants with valid weekly sleep and valid sleep measured the night prior to cognitive testing.

	Response time (ms)	Response accuracy (proportion correct)
**Working memory task**^a^		
1-Back	409.1 ± 73.6	0.98 ± 0.02
2-Back	522.8 ± 133.8^b^	0.95 ± 0.06^b^
3-Back	538.0 ± 150.6^b,c^	0.87 ± 0.08^b,c^
**Visual attention task**^a^		
Valid cue	307.2 ± 31.9	0.94 ± 0.05
Invalid cue	360.2 ± 40.6^d^	0.91 ± 0.09^d^
No cue	387.8 ± 45.6^d,e^	0.96 ± 0.04^d,e^

Results presented as median ± interquartile range due to skewed distributions.

^a^Differences evaluated using mixed effect regression of transformed variables with Bonferroni post hoc correction for multiple comparisons.

^b^Significantly different than 1-back load.

^c^Significantly different than 2-back load.

^d^Significantly different than valid cue presentation.

^e^Significantly different than invalid cue presentation.

Response times for all three memory loads were positively correlated with one another (all *p* ≤ 0.01; Table S1 in the Supplementary Information), suggesting those who responded rapidly on lower memory load also did so on higher memory loads. Correlations between response accuracies were less consistent, with a positive correlation only between 2-back and 3-back accuracies (*p* ≤ 0.05). We found limited correlation between response time and accuracy for each cognitive load, with the exception of a negative correlations for the 3-back load (*p* < 0.05), suggesting those with faster responses were also more accurate for this load.

#### Visual attention task

Visual attention was evaluated using a Posner cue-target paradigm task^26–28^. Participants were instructed to focus on a cross centred on the screen between two rectangles and indicate whether the target stimulus (an asterisk) appeared in the left or right rectangle. The task consisted of a pre-determined sequence of three possible cue presentations: “valid cue”, “invalid cue”, and “no cue”. “Valid cue” presentation occurred when a rectangle frame thickened (cue) as the target stimulus appeared at its centre. “Invalid cue” presentation occurred when a rectangle frame thickened as the target stimulus appeared inside the opposite rectangle. “No cue” presentation was the absence of either rectangle frame thickening during target stimulus appearance^28,29^.

Response time and accuracy on the visual attention task also varied significantly across conditions (all *p* < 0.001, Table 1). However, trends across cue presentations for response time differed from those of response accuracy. The no cue presentation elicited the slowest but most accurate responses. During the valid cue presentation, responses were fastest, but accuracy was intermediate between no cue and invalid cue presentations. On the other hand, during the invalid cue, responses were least accurate, but response times were intermediate between the valid and no cue presentation.

Response times for each cue presentation in the visual attention task were all highly positively correlated to one another (all *p* < 0.001, Table S2 in the Supplementary Information). Similarly, response accuracies of all cue presentations were also all positively correlated (all *p* ≤ 0.01). Taken together, these results suggest that those who responded rapidly and accurately on one cue presentation did so on others. As in the working memory task, correlations between response time and response accuracy were inconsistent by cue presentation. While response time was directly correlated to response accuracy during the invalid cue presentation (*p* = 0.03), it was inversely correlated to accuracy during the no cue presentation (*p* < 0.001) and unrelated to accuracy for the valid cue presentation.

### Associations between sleep and cognitive measures

Associations between cognitive measures and free-living sleep parameters were assessed using linear regression models. Presence or absence of a clinical diagnosis of ADHD and weekly videogame usage were determined via self-report and included in all regression models since the presence of ADHD and extensive videogame use have previously been associated with cognitive performance in visuospatial tasks^30,31^. Response times and accuracies on higher cognitive loads of the working memory task (i.e. 2-back and 3-back response times) were adjusted for analogous measures on the 1-back task^32,33^. Similarly, response times and accuracies of the invalid and no cue conditions of the visual attention task were adjusted for the analogous measures on the valid cue condition.

**Figure 1:**
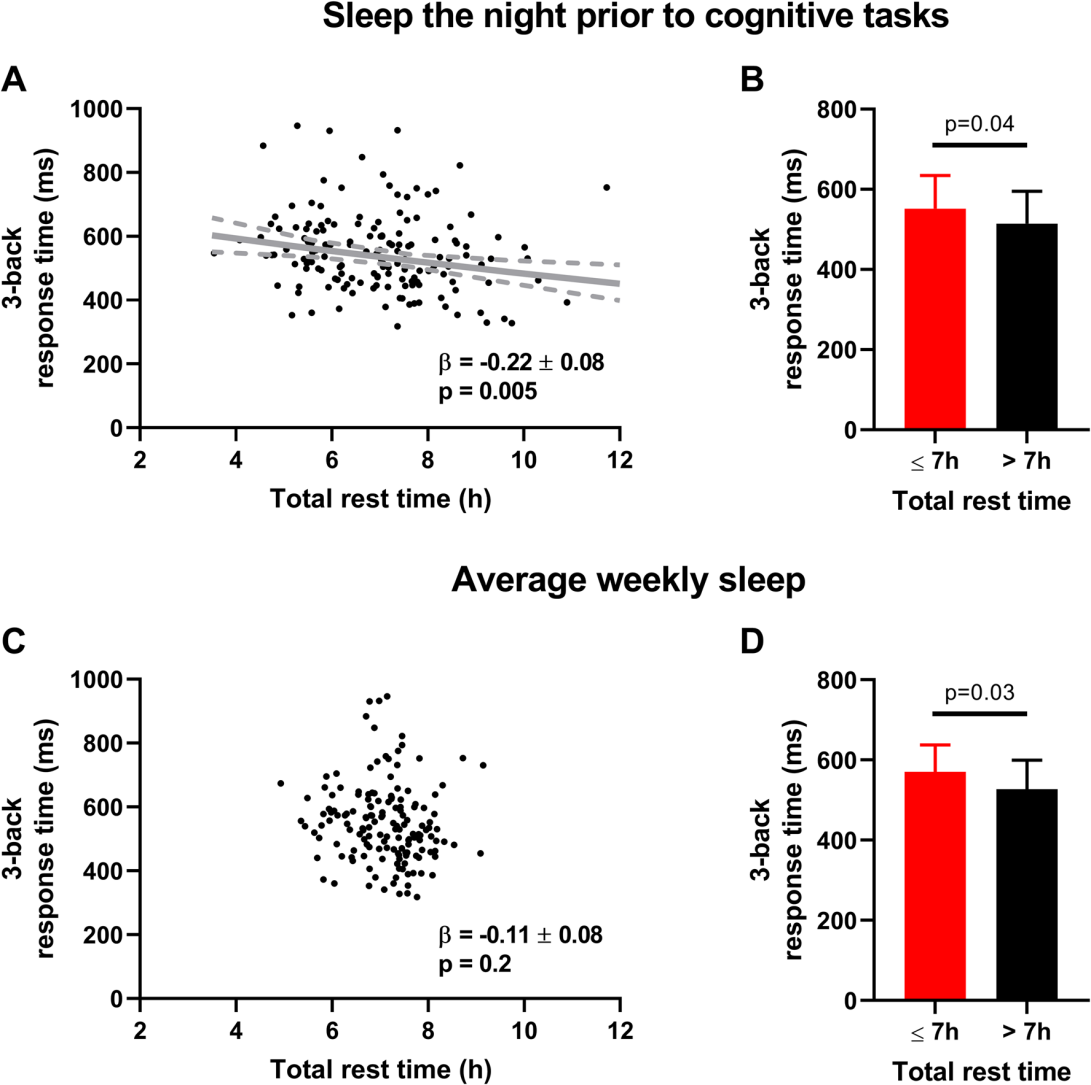
Relationship between response time on the most difficult (3-back) work memory load and total rest time. (**A**) The solid grey line demonstrates the inverse correlation between total rest times the night prior to the cognitive task and 3-back response times. Broken grey lines indicate the 95% confidence intervals. (**B**) Participants with 7 h or less total rest time (in red, N = 82) had longer response times than those with greater than 7 h (in black, N = 78). (**C**) Average weekly total rest time did not correlate with 3-back response times. (**D**) Participants that average 7 h or less daily total rest time over the week (N = 66) had longer response times than those that averaged greater than 7 h (N = 94). All comparisons adjusted for clinical diagnosis of attention deficit hyperactivity disorder, reported weekly video game use, and 1-back response times. Response times were log-transformed prior to analysis due to skewed distributions; inverse transformation was applied for displayed in (**A**); bars and error bars in (**B**) and (**D**) are medians and interquartile ranges. β, standardized beta ± standard error.

We first explored the association between working memory and sleep parameters measured the night prior to cognitive testing and observed *p* values below 0.05 for the positive association between sleep efficiency and 2-back response time and the negative associations between total rest and sleep times and 3-back response times (Table 4). Next, we examined the association between working memory and weekly averages of sleep parameters and found no associations with *p* values below 0.05 (Table 4). To control for possible type 1 error, we performed a Benjamini–Hochberg analysis of the 40 comparisons summarized in Table 4 with a false discovery rate (Q) of 0.25, and determined that only the association between rest duration the night before cognitive testing and 3-back response time (*p* = 0.005) fell below the critical *p* value of 0.006. This suggests that less time in bed acutely, the night prior to the working memory task, was significantly associated with longer response times only on the most difficult cognitive load (Fig. 1A), although no such relationship existed for average weekly time in bed (Fig. 1C).

**Table 4:** Results of linear regression between sleep parameters and response times and accuracies on the short-term working memory task.

	2-Back response time (ms)	2-Back response accuracy (proportion correct)	3-Back response time (ms)	3-Back response accuracy (proportion correct)
	β [95% CI] (*p*)	β [95% CI] (*p*)	β [95% CI] (*p*)	β [95% CI] (*p*)
**Sleep measures of the night prior to short-term memory task**				
Total rest time (h)	− 0.03 [− 0.18, 0.11] (0.7)	− 0.02 [− 0.18, 0.15] (0.9)	− 0.22 [− 0.38, − 0.07] (0.005)*	0.04 [− 0.12, 0.20] (0.6)
Total sleep time (h)	0.02 [− 0.13, 0.16] (0.8)	− 0.06 [− 0.22, 0.10] (0.5)	− 0.19 [− 0.34, − 0.03] (0.02)	0.07 [− 0.09, 0.23] (0.4)
Sleep efficiency (%)	0.15 [0.01, 0.29] (0.04)	− 0.15 [− 0.31, 0.00] (0.06)	0.03 [− 0.13, 0.18] (0.8)	0.09 [− 0.07, 0.25] (0.3)
WASO (min)	− 0.13 [− 0.27, 0.01] (0.06)	0.13 [− 0.03, 0.29] (0.1)	− 0.12 [− 0.28, 0.04] (0.1)	− 0.08 [− 0.24, 0.08] (0.3)
**Weekly sleep measures**				
Total rest time (h/night)	− 0.01 [− 0.15, 0.13] (0.9)	− 0.06 [− 0.22, 0.10] (0.5)	− 0.11 [− 0.26, 0.05] (0.2)	0.11 [− 0.05, 0.26] (0.2)
Total sleep time (h/night)	0.04 [− 0.10, 0.18] (0.6)	− 0.04 [− 0.20, 0.12] (0.6)	− 0.07 [− 0.23, 0.09] (0.4)	0.13 [− 0.03, 0.28] (0.1)
Sleep efficiency (%)	0.10 [− 0.04, 0.24] (0.2)	− 0.004 [− 0.16, 0.16] (0.96)	0.06 [− 0.10, 0.21] (0.5)	0.05 [− 0.11, 0.20] (0.6)
WASO (min/night)	− 0.10 [− 0.24, 0.04] (0.2)	− 0.06 [− 0.22, 0.10] (0.5)	− 0.08 [− 0.24, 0.07] (0.3)	− 0.05 [− 0.20, 0.11] (0.6)
Total rest time variability (h)	0.01 [− 0.13, 0.15] (0.9)	− 0.04 [− 0.20, 0.12] (0.7)	0.15 [− 0.01, 0.30] (0.07)	− 0.05 [− 0.21, 0.11] (0.5)
Total sleep time variability (h)	0.002 [− 0.14, 0.14] (0.98)	− 0.02 [− 0.18, 0.14] (0.8)	0.13 [− 0.03, 0.28] (0.1)	− 0.07 [− 0.22, 0.09] (0.4)

β, standardized beta value; CI, confidence interval; WASO, wake time after sleep onset.

*Significant after Benjamini–Hochberg analysis of all comparisons with 0.25 false discovery rate. All regressions adjusted for clinical diagnosis of attention deficit hyperactivity disorder and reported weekly video game use. 2-back and 3-back response time additionally adjusted for 1-back response times. 2-back and 3-back response accuracy additionally adjusted for 1-back response accuracy. Response times, response accuracies, sleep efficiency, total sleep time variability, and total rest time variability were transformed prior to analysis due to skewed distributions.

In an additional, exploratory analysis of the relationship between total rest time and working memory, we tested whether participants with a total rest time of 7 h or less performed differently on the task than those with greater than 7 h. The threshold value of 7 h of total rest time was selected since it was the group mean for the participants in this study and previously used as an indicator of short sleep^34^. We found that the 82 students with a rest duration of 7 h or less the night prior to cognitive testing had longer 3-back response times on than the 78 students with a rest duration of greater than 7 h (median ± interquartile range: 551.7 ± 148.1 vs. 514.5 ± 138.2 ms, *p* = 0.04; Fig. 1B, Table S3). We also found that the 66 participants who averaged 7 h or less total rest time over the week had longer 3-back response times than the 94 participants who did not (570.3 ± 135.7 vs. 511.3 ± 143.7 ms, *p* = 0.03; Fig. 1D, Table S3). Thus, results of the categorical analysis support the finding that short, acute total rest time prior to cognitive testing is associated with longer response times on the most difficult cognitive task. Analyzing the data in a categorical fashion also allowed us to detect a similar difference in 3-back response time between those with shorter and longer weekly rest time.

Surprisingly, we found no associations between response times on the visual attention task and sleep parameters measured the night prior to cognitive testing or over the entire week (Table 5). There were also no differences in the visual attention task performance of participants with 7 h or less total rest time compared to those with greater than 7 h (Table S4).

**Table 5:** Results of linear regression between sleep parameters and response times and accuracies on the visual attention task.

	Invalid cue response time (ms)	Invalid cue response accuracy (proportion correct)	No cue response time (ms)	No cue response accuracy (proportion correct)
	β [95% CI] (*p*)	β [95% CI] (*p*)	β [95% CI] (*p*)	β [95% CI] (*p*)
**Sleep measures of the night prior to short-term memory task**				
Total rest time (h)	− 0.05 [− 0.14, 0.04] (0.3)	0.01 [− 0.09, 0.11] (0.8)	0.04 [− 0.05, 0.13] (0.3)	− 0.08 [− 0.24, 0.09] (0.4)
Total sleep time (h)	− 0.06 [− 0.15, 0.03] (0.2)	0.02 [− 0.09, 0.12] (0.8)	0.03 [− 0.06, 0.12] (0.5)	− 0.07 [− 0.23, 0.09] (0.4)
Sleep efficiency (%)	− 0.05 [− 0.14, 0.04] (0.3)	− 0.01 [− 0.11, 0.09] (0.9)	− 0.02 [− − 0.11, 0.07] (0.6)	− 0.02 [− 0.18, 0.14] (0.8)
WASO (min)	0.02 [− 0.07, 0.11] (0.7)	− 0.01 [− 0.11, 0.10] (0.9)	0.04 [− 0.05, 0.13] (0.4)	− 0.02 [− 0.18, 0.14] (0.8)
**Weekly sleep measures**				
Total rest time (h/night)	0.01 [− 0.08, 0.10] (0.8)	− 0.01 [− 0.11, 0.09] (0.9)	0.02 [− 0.07, 0.10] (0.7)	0.04 [− 0.12, 0.19] (0.7)
Total sleep time (h/night)	− 0.02 [− 0.11, 0.07] (0.7)	0.03 [− 0.07, 0.13] (0.6)	0.01 [− 0.08, 0.10] (0.8)	0.02 [− 0.14, 0.18] (0.8)
Sleep efficiency (%)	− 0.07 [− 0.16, 0.02] (0.1)	0.07 [− 0.03, 0.17] (0.2)	− 0.01 [− 0.1, 0.08] (0.8)	− 0.05 [− 0.21, 0.11] (0.6)
WASO (min/night)	0.07 [− 0.02, 0.16] (0.1)	− 0.07 [− 0.17, 0.03] (0.2)	0.01 [− 0.08, 0.09] (0.9)	0.04 [− 0.12, 0.20] (0.6)
Total rest time variability (h)	− 0.001 [− 0.09, 0.09] (0.98)	0.03 [− 0.07, 0.13] (0.6)	− 0.07 [− 0.16, 0.01] (0.1)	0.03 [− 0.13, 0.18] (0.7)
Total sleep time variability (h)	− 0.01 [− 0.1, 0.08] (0.8)	0.05 [− 0.05, 0.15] (0.3)	− 0.06 [− 0.15, 0.02] (0.2)	− 0.003 [− 0.16, 0.16] (0.97)

All regressions adjusted for clinical diagnosis of attention deficit hyperactivity disorder and reported weekly video game use. Invalid cue and no cue response time additionally adjusted for valid cue response times. Invalid cue and no cue response accuracy additionally adjusted for valid cue response accuracy. Response times, response accuracies, sleep efficiency, total sleep time variability, and total rest time variability were transformed prior to analysis due to skewed distributions.

β, standardized beta value; CI, confidence interval; WASO, wake time after sleep onset.

## Discussion

We explored the association between objectively measured free-living sleep and working memory and visual attention tasks in healthy adolescents since prior studies conducted in clinical settings have shown that sleep restriction affects these cognitive functions^35^. We found that shorter time in bed the night prior to the cognitive testing was negatively associated with performance on the most challenging short-term memory load, indicating that acute short sleep can affect short-term working memory, even in a healthy population measured in a free-living setting. We did not observe a similar continuous association between weekly rest duration and short-term working memory, despite short average sleep duration and time in bed over the week. However, we did find that participants with rest durations of 7 h or less, measured either acutely on the night prior to cognitive testing or averaged over the week, had longer response times during the most difficult short-term memory load than those with greater than 7 h. Taken together with the considerable intra-participant night-to-night variation in sleep and rest duration, this suggests that compensatory bouts of shorter and longer sleep over the week may obscure associations between sleep duration and working memory and may require a larger sample size to detect a correlation based on continuous measures. Despite strong previous evidence to the contrary from clinical studies, we did not observe any association between sleep parameters and performance on the visual attention task. This suggests that the task lacked complexity, the variability of free-living sleep parameters was too high, or both.

Sleep curtailment has been shown to have a deleterious effect on cognitive function both in laboratory studies^9,35^ and actigraphy-based sleep assessment^15^. For example, adolescents aged 15–19 years randomly assigned to seven nights of sleep restriction, i.e. 5 h of sleep opportunity, had lower sustained attention, working memory, executive function, and processing speed compared to those assigned to 9 h of sleep opportunity^9^. Similarly, four nights of partial sleep restriction (6–6.5 h/night) significantly reduced information processing speed compared to four nights of sleep extension (10–10.5 h/night) for adolescents with a mean age of 16.9 years^36^. Interestingly, a previous study of younger adolescents (aged 6–13 years) found that shorter actigraphy-measured sleep averaged over three nights was associated with reduced performance only on the highest memory load of on an n-back task^15^. Other studies have also reported that reduced sleep duration might be particularly important for more demanding tasks that require enhanced working memory capacity and concentration^17^. It should also be noted that most interventional studies define study arms according greater or lesser sleep opportunity while most self-reported measures rely either on a typical time-in-bed or the difference between bedtime and rise time, and do not take into account nightly awakenings since awakenings are generally difficult to measure by report^37^. Perhaps the closest actigraphy-derived variable to these measures is the total rest time, which, unlike total sleep time, does not subtract minutes of awakenings. Thus, our finding that rest time the night before cognitive testing is negatively associated with performance on the most challenging memory load of the n-back task is in-line with these previous findings and demonstrates that the deleterious effects of short sleep on demanding tasks of memory are also present in the free-living setting for older adolescents.

Although our finding that shorter rest duration the night prior to testing was associated with slower responses at the highest cognitive load of the memory task is in line with that from another actigraphy study of adolescents^15^, our results differed in that we did not detect an association between sleep quality and working memory performance. However, the mean age of the participants in that study (9.9 ± 1.9 years) was much younger and the age range (6.9–13.3 years) was much broader than the current study. The authors also noted that age associated positively with sleep duration and negatively with sleep efficiency^15^, indicating that younger participants likely slept longer than 8.2 ± 0.6 h/night with an efficiency below 86.5 ± 5.1%, the reported averages for each parameter. A recent study of healthy adults with a mean age closer to that of our participants (approximately 21 years) found no association between subjective sleep quality and working memory^38^. The authors suggest that the lack of association may have been due to a ceiling effect of studying a healthy population with limited prevalence of disorders known to cause sleep disturbances, which is also the case in the present study. These observations suggest that, compared to younger populations, free-living sleep quality may play a lesser role than sleep duration in the working memory task performance of healthy older adolescents and young adults.

We did not detect any significant continuous associations between working memory and weekly averages of sleep duration or quality. This lack of association was surprising as we expected observations of sleep patterns over a longer duration would have greater associations with cognitive function than acute sleep observations, but we have considered several potential explanations. The pervasive short sleep in our study sample, where over 88% averaged less than the recommended 8 h time-in-bed over the week, may have resulted in a floor effect that made it difficult to detect associations between weekly sleep duration and cognitive function. The absence of cognitive results following a period of recommended sleep opportunity for comparison, as is common in most clinical interventions, also complicates the interpretation of the results, since both sleep needs^39,40^ and performance on cognitive tasks^41^ are likely to be individualized. However, it should also be noted that due to biological changes during puberty^42^, older adolescents may be able to remain awake longer and may be less likely to notice to sleep deficits than younger adolescents and adults since they accumulate homeostatic sleep pressure more slowly^43,44^ and are less sensitive to its effects^44,45^.

On the other hand, the minimal associations between free-living sleep and cognitive function may have been due to a ceiling effect in the performance on the cognitive tasks related to the peak in the cognitive function that reportedly occurs in young adulthood, close to the age range of our generally healthy, older adolescent population. Working memory has been reported to peak in young adulthood^38,41,46,47^. In support of this, the responses on all cognitive loads of the working memory task in the current study were faster and more accurate than in a previous study of male participants with a slightly older mean age (28 ± 4 years) tested with the same paradigm in our laboratory^48^. In contrast, performance on visual attention tasks may be less related to age^49^. However, in the current study we observed response times and accuracies that were similar to those of a group of male Norwegian participants within the same age range (17.9 + 0.9 years) undergoing the same visual attention task^29^, suggesting a similar level of motivation and performance. Thus, we cannot determine whether the cognitive tasks used in our study may have lacked the sensitivity to assess the effects of free-living sleep on working memory and visual attention of our generally healthy, older adolescent population and the topic warrants further study.

Another potential explanation for the limited association between weekly sleep and cognitive function was that the participants were able to compensate for low sleep duration over the week. The high intra-individual variation in sleep duration is indicative of compensatory nights of shorter and longer sleep over the week, which may obscure associations between weekly sleep parameters and cognitive function. However, irregular sleep patterns have also been shown to be associated with poorer academic performance in younger children^50^ and college-aged students^51^. Collectively, these observations suggest that any compensatory benefit of a highly varied sleep schedule on cognitive function are likely outweighed by the negative impacts on cognition. The participants may also have compensated for short sleep with caffeine intake, which we did not control for and is reportedly widespread amongst Icelandic adolescents^52^. These potential compensatory behaviours may have led to a more subtle relationship between free-living sleep and cognitive function than those observed during clinical trials with greater control of diet and sleep schedule.

The high night-to-night variability in rest and sleep duration amongst participants in this study also suggests that a larger sample size may be required to detect a correlation based on continuous measures of free-living rest or sleep duration and cognitive function in older adolescents. The standardized beta coefficients of the associations between 3-back response time and total rest times for the night prior to cognitive testing (β = − 0.22 ± 0.08; standardized β ± standard error) and averaged over the week (β = − 0.11 ± 0.08) both indicated inverse relationships and did not significantly differ when tested statistically. However, only total rest time measured on the night prior to cognitive testing was significantly correlated with 3-back response times. Dichotomizing a continuous variable can increase the statistical power to detect differences. Employing this strategy, we found that 3-back response times were shorter for participants with total rest time above versus below the 7-h group average, independent of the sleep measurement duration. Taken together, these observations suggest that, although sleep measured over both durations may have similar relationships to working memory task performance, in our sample, sleep measured acutely before testing had a stronger relationship to 3-back response time and more subjects are likely needed to increase statistical power enough to detect a similar continuous association between 3-back response time and weekly rest time.

As with the limited association between free-living sleep and working memory, the absence of a relationship between free-living sleep and visual attention likely stems from a confluence of factors, including many discussed previously, such as the likelihood of compensatory behaviours, limitations in statistical power, and the floor effect of wide-spread short sleep. Prior research on the relationship between sleep and attention has demonstrated that errors increase with continued wakefulness^53^. Despite average short sleep both over the entire week and acutely before the cognitive task, < 8% of participants spent less than 5 h in bed the night before the task, a duration of sleep opportunity previously used in studies of acute sleep restriction in adolescent^9^. Thus, although the limited rest duration may have affected performance of the most complex working memory task, greater deprivation may be required for detectable degradation of attention task performance, particularly when considering the size of our sample relative to the inter-participant variation in time-in-bed (1.4 h). Attention task errors have also been shown to increase with task duration^54^, and short sleep has greater impact with increasing task complexity^17^. The duration of our visual attention task (9 min) was short and thus, a longer and/or more complex task may be needed to better demonstrate the relationship between free-living sleep and visual attention.

Most previous studies of the relationship between sleep and cognition have used controlled sleep interventions on a smaller sample in a laboratory setting. Unlike inpatient sleep assessments, free-living sleep measurements benefit from monitoring sleep patterns in a familiar environment and allow subjects to maintain typical daily routines and habits. Our chosen method of measuring free-living sleep, wrist actigraphy, is less sensitive to subjective bias and has greater accuracy to detect sleep duration^55^ and awakenings^56^ than self-report methods when compared to the gold standard—polysomnography. However, actigraphy is more difficult to administer than questionnaire-based self-report, which limited our sample size, although it is larger than most clinical sleep studies and representative of Reykjavík students in this age group during the study period (1382 17-year-olds in 2017)^57^.

Although wrist actigraphy has been shown to have high accuracy and sensitivity compared to polysomnography, its specificity is limited^58^. Further, our analysis focused on the primary nightly sleep period since the parameters of the automated detection algorithm may have been insufficient to detect naps and there is no accepted criterion for scoring actigraphy-assessed naps^59^ and the automated sleep detection algorithm may have been inadequate to detect naps. Participants were only explicitly instructed to log primary sleep periods and may not have thought to include naps in the diary. Thus, we were unable to determine whether short periods of inactivity outside of the primary sleep period were naps due to a lack of a validated automated method or confirmatory log. Exclusion of naps could have affected our results, as napping has been shown to be a prominent behaviour among this age group and is associated with both shorter and more disrupted night-time sleep^59^.

It was not feasible to control the time of day or day of week for cognitive testing due to the highly varied schedules of the participants in this age group, which is a considerable limitation. However, all tests were conducted on weekdays in the afternoon (starting from 12:30–19:00, with a mean start time of 15:43 ± 1.3 h) and in preliminary analyses, we found no correlation between cognitive response times and time of day or day of the week for testing. Similarly, we found no theoretical basis for a sex-based difference in the relationship between sleep and cognitive responses in previous literature^60^ or preliminary analyses. Thus, final regression models were not statistically adjusted for these variables.

Finally, this was an exploratory analysis of a secondary outcome for an ongoing longitudinal study of Icelandic youth. Thus, it was not powered to detect a pre-specified outcome and the homogeneity of the study sample may limit the generalizability of the results. However, the results provide a basis for future study design in broader and more diverse populations. The cross-sectional design of the analysis and the absence of comparable cognitive data following a period of “sufficient” sleep makes it difficult to determine cause and effect.

### Conclusions

Our study of the relationship between free-living sleep and cognitive function in generally healthy, older adolescents demonstrated that shorter time in bed the night prior to the cognitive testing was negatively associated with performance on the most challenging short-term memory load. However, despite a short and varied sleep duration over the week, we did not find a clear, continuous association between weekly sleep measures and short-term working memory or visual attention as hypothesized. Taken together, these results suggest that the considerable intra-participant night-to-night variation in sleep duration observed with free-living sleep of older adolescents may result in a relationship between sleep and cognitive task performance that is less stark than those observed with laboratory-imposed sleep restriction. Nevertheless, the negative impact of shorter acute sleep on difficult tasks of working memory appears to persist even in a generally healthy population measured in a free-living setting.

## Methods

### Participants

**Figure 2:**
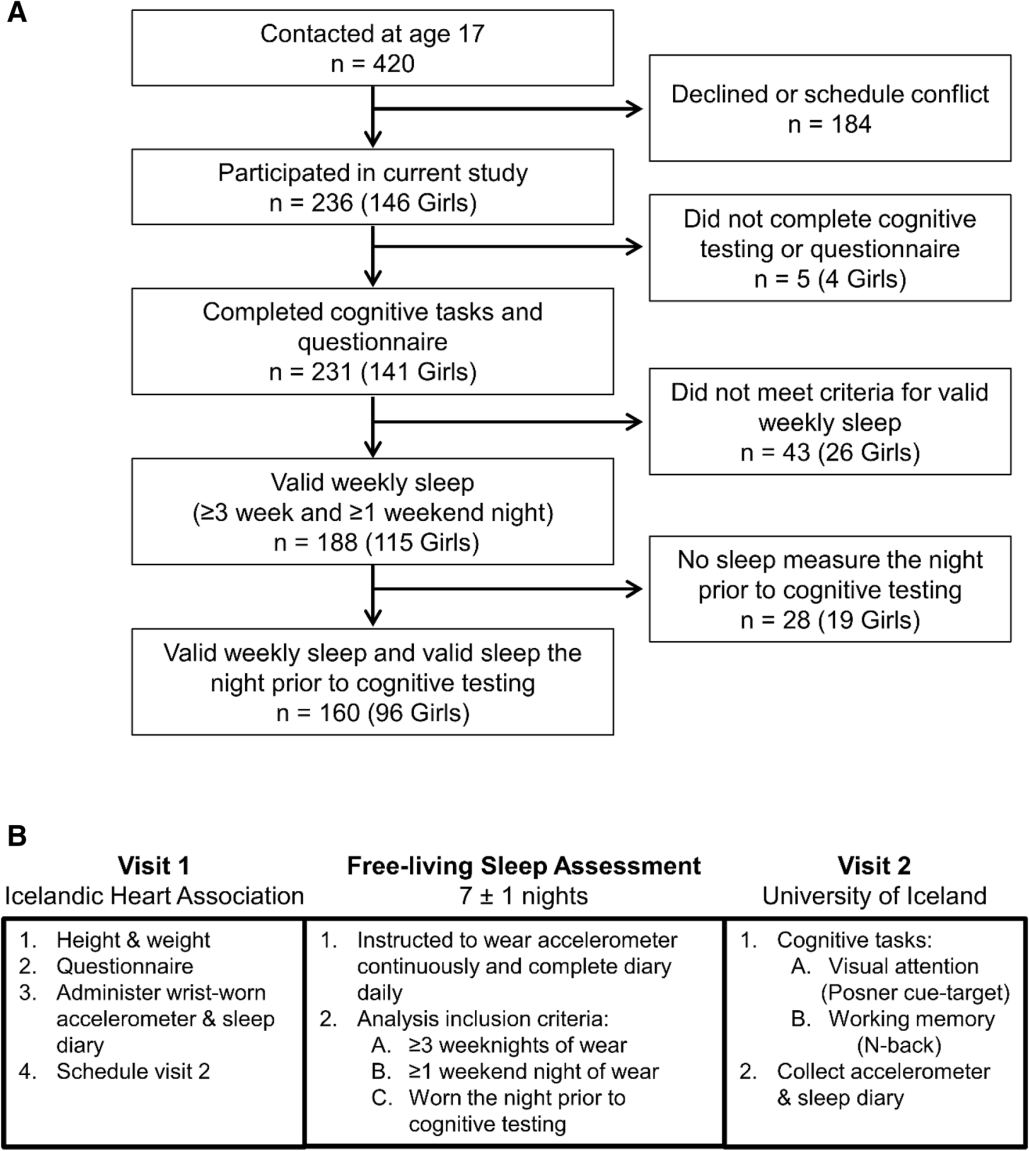
(**A**) Participant flowchart. (**B**) Schematic representation of the study procedure.

We were able to contact 420 participants who had participated in previous waves of the study and 236 (146 girls) agreed to participate (56% participation rate) and 231 (61% girls) had height and weight measured and completed the cognitive tasks and the questionnaire (Fig. 2A). Free-living sleep was measured for one week with wrist actigraphy, and 188 participants (62% girls) also met the requirements for valid sleep measurements over the week (≥ 3 weeknights and ≥ 1 weekend night). However, due to schedule conflicts or non-compliance, 28 participants with valid weekly sleep measures did not have a sleep measurement the night prior to cognitive testing. The remaining 160 participants with valid sleep measures over the week and the night prior to cognitive testing were included in the analysis.

### Data collection

We collected the data from early February until early May of 2017. Each participant had two visits separated by 7 ± 1 days on average (min = 6, max = 17, median = 7). The first visit occurred at the Icelandic Heart Association where participants completed a tablet-based questionnaire, weight and height were measured by trained research staff, and participants were provided with a wrist-worn accelerometer. The second visit occurred at the University of Iceland where participant performed cognitive tasks and returned the accelerometer (Fig. 2B).

The study was conducted in agreement with the guidance provided in the Declaration of Helsinki and the National Bioethics Committee and the Icelandic Data Protection Authority approved the study (Study number: VSNb2015020013/13.07). Written informed consent was obtained from participants or guardians of underage participants.

### Sleep measures

Participants were asked to wear a small (3.8 cm × 3.7 cm × 1.8 cm), lightweight (27 g) accelerometer (model GT3X+, Actigraph Inc., Pensacola FL) on their non-dominant wrist for one entire week. Tri-axial accelerometer data recorded at 80 samples/second was subsequently filtered and aggregated into one-minute activity counts with Actilife software version 6.13.0 (Actigraph Inc., Pensacola, FL, USA). Periods of sixty or more consecutive minutes of zero counts on all axes were identified as non-wear by customized programs in Matlab version R2016B (Mathworks Inc., Natick, MA). Each night was considered valid if the device was worn for ≥ 14 h from 12 to 12 noon the next day and the longest detected sleep period beginning within that interval was analysed. As detailed previously^23^, to make full use of software-based editing functions, nightly sleep periods and awakenings were first detected in the Actilife software with the Sadeh algorithm validated for adolescents^61^. Each software-detected rise- and bedtime was then visually confirmed or adjusted by two trained scorers, using participant daily sleep diaries only when necessary and available. The total rest time, total sleep time, sleep efficiency, and WASO were computed for each night of sleep. Weekly averages of all sleep parameters and the weekly variability of rest and sleep duration were computed and used in regression analyses for participants with valid data on ≥ 3 weeknights (Sunday-Thursday) and ≥ 1 weekend night (Friday, Saturday, and nights prior to school holidays), based on guidelines presented in a recent systematic review of standards for accelerometer data collection^62^ and in concordance with the criteria used in our previous analyses^23,25,63^.

### Cognitive measures

Short-term working memory and visual attention were assessed with previously validated software-based tasks in E-prime 2.0 (standard version, Psychology Software Tools, Inc., Pittsburgh, PA). Tasks were administered on a 13.3^″^ laptop computer in a quiet corner while wearing noise-cancelling headphones. Before each task, participants were given verbal instructions and provided with a short practice session to make sure they understood the instructions. Response time and response accuracy, the proportion of correct responses were the outcome measure for each stimulus category^28,29,64^. Erroneous responses consisted of commission errors (i.e. wrong button pushed) and omission errors (incorrect rejections and responses provided ≤ 149 ms after target appearance)^29^.

#### Short-term memory task

**Figure 3:**
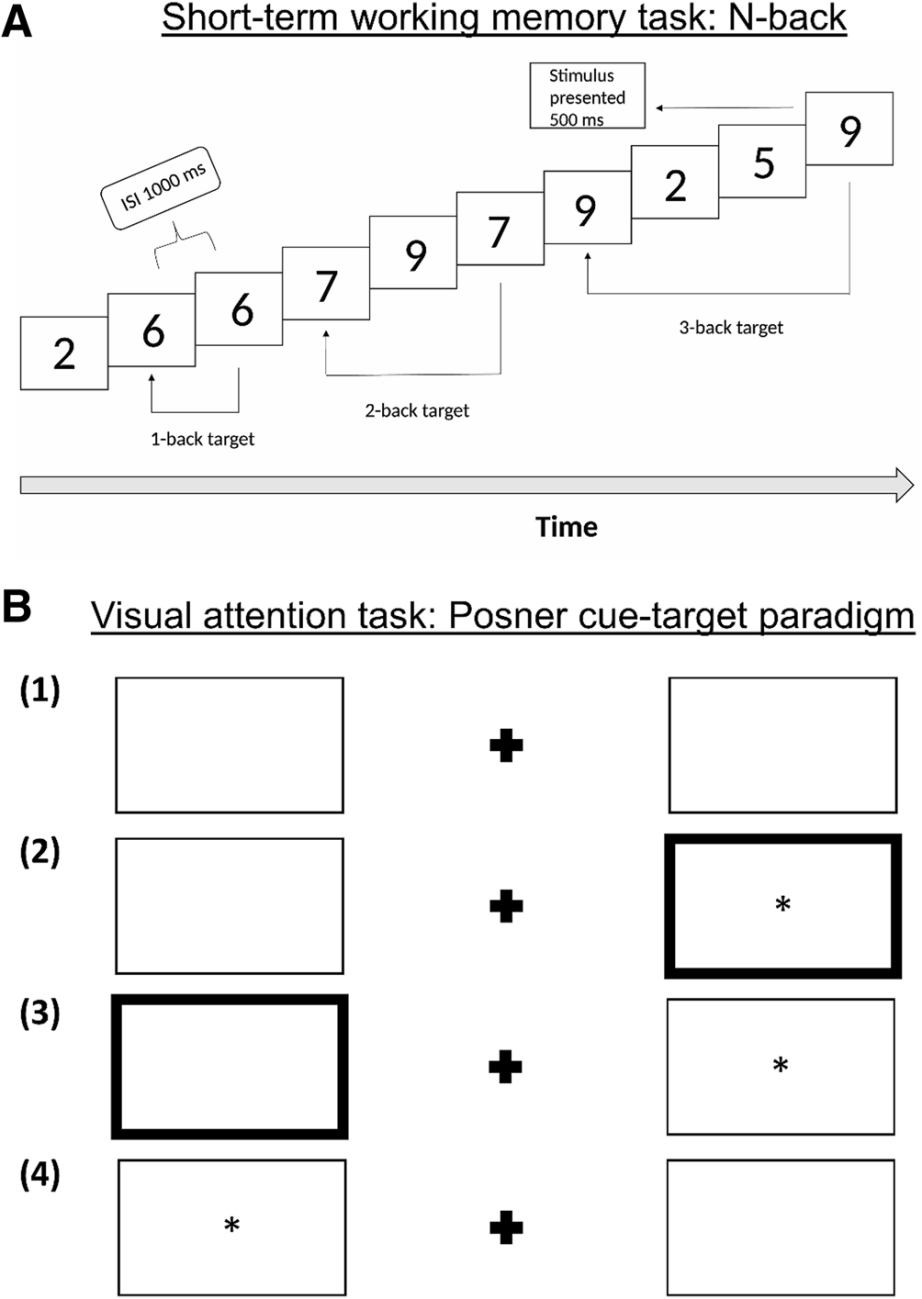
Schematic representation of the cognitive tasks. (**A**) The N-back task for working short-term memory. Sixty-three digits were presented one at a time for each session of one working memory load condition, which varied from 1-back (least difficult) to 3-back (most difficult). Each stimulus was presented for 500 ms with an inter-stimulus-interval of 1000 ms. (**B**) The Posner cue target task for visual attention. (1) Screen appearance prior to cue presentation and stimulus appearance—central cross with rectangles to the right and left. (2) Valid cue presentation—borders thicken (cue) prior to target stimulus appearance inside the rectangle. (3) Invalid cue presentation—target stimulus appears opposite to cue rectangle. (4) No cue presentation prior to target stimulus appearance.

A numeric-based version of the n-back task was used to assess short-term working memory (Fig. 3A). Participants were instructed to quickly and accurately press the spacebar if the current digit was the same as the one presented n positions back in the sequence, where n varied from 1–3, with higher numbers representing greater working memory load. Each digit was presented for 500 ms with an interstimuli-interval of 1000 ms. The task was comprised of three sessions with the working memory load condition increasing from 1-back to 3-back^65^. Sixty-three digits were presented for each session with ten correct answers.

#### Visual attention task

Visual attention was evaluated using a Posner cue-target paradigm task^26–28^ with a pre-determined sequence of three possible cue presentations: “valid cue”, “invalid cue”, and “no cue” (Fig. 3B). Each stimulus was presented for 500 ms with interstimuli-intervals between 600 and 1400 ms. A total of 336 target stimuli were presented, 224 (67%) with a valid cue, 56 (17%) with an invalid cue, and 56 (17%) with no cue^29^. The sequence and timing of cue and target stimulus presentations were the same for all participants.

### Covariates

Participants self-reported videogame use and presence or absence of a clinical diagnosis of ADHD on the questionnaire during the initial visit. Videogame use was reported using a seven-point scale with the following response options presented separately for weekdays and weekends: 1 = “none”, 2 = “about ½ h”, 3 = “1 up to 2 h”, 4 = “2 up to 3 h”, 5 = “3 up to 4 h”, 6 = “4 to 5 h” and 7 = “more than 5 h”. A weighted average was computed from the weekday and weekend responses using the midpoints of each response category or 0.5 h/day for the “about half an hour” response option and 5.5 h/day for the “more than 5 h” response option (only four participants selected this response). Participants reported clinical diagnosis of ADHD by answering yes or no to the question “Have you been clinically diagnosed with ADHD?”.

### Statistical analyses

The distributions of all variables were examined for normality to determine whether transformations were needed prior to the use of parametric statistics. Response times, response accuracies, sleep efficiency, and the weekly variability in rest and sleep duration all had asymmetric, non-normal distributions and are reported as median ± interquartile range. All other variables are reported as mean ± standard deviation. Correlations between response times and response accuracies were assessed using the non-parametric Spearman method. Prior to outlier detection and regression analyses, log-transformation was applied to response time variables and the variability in rest and sleep duration and arcsine transformation (i.e. arc-sine of the square root of the proportion variable) was applied to response accuracies and sleep efficiencies. Values that exceeded 3 standard deviations above or below the mean were considered outliers and excluded. Mixed-effect models with post hoc tests with Bonferroni correction for multiple comparisons were performed to evaluate differences in transformed response times and response accuracy across stimuli on the visual attention task and across cognitive load on the working memory task. Linear regression models adjusted for baseline cognitive responses (i.e. 1-back or valid cue response time or accuracy), clinical diagnosis of ADHD, and time spent playing video games were used to assess associations between sleep parameters and cognitive task performance, with results reported as standardized β with 95% confidence intervals. Two sets of regression analyses were performed, the first with sleep parameters from the night prior to cognitive testing as predictor variables and the second with average weekly sleep parameters and sleep variability parameters as predictor variables. Categorical analyses were adjusted for baseline cognitive responses, clinical diagnosis of ADHD, and reported weekly video game use. *p* values less than 0.05 were considered significant unless otherwise stated. Analyses were conducted using R (v3.4.2, https://www.r-project.org/) and GraphPad Prism (v7, La Jolla, CA).

## Supplementary information

Supplementary file1

## Data Availability

Datasets for the current study are available from the corresponding author upon reasonable request.
