# Supplementary material for: Association between free-living sleep and memory and attention in healthy adolescents
Source: Sci Rep. 2020 Oct 9;10:16877. doi: 10.1038/s41598-020-73774-x (PMC7547704; doi:10.1038/s41598-020-73774-x)
Supplement: Supplementary file 1 — Supplementary file1 [file 41598_2020_73774_MOESM1_ESM.docx]

**Supplementary Information:**

**Association Between Free-living Sleep and Memory and Attention in Healthy Adolescents**

Runa Stefansdottir,^a^ Hilde Gundersen,^b^ Vaka Rognvaldsdottir,^a^ Alexander S. Lundervold,^c,d^ Sunna Gestsdottir,^a^ Sigridur L.Gudmundsdottir,^a^ Kong Y.Chen,^e^ Robert J.Brychta,^e,1^ Erlingur Johannsson^a,b,1*^

**Affiliations:** ^a^ Centre for Sports and Health Sciences, University of Iceland, Reykjavik, Iceland: ^b^ Department of Sport, Food and Natural Sciences, Western Norway University of Applied Sciences; ^c^ Department of Computer Science, Electrical Engineering, and Mathematical Sciences, Western Norway University of Applied Sciences, Bergen, Norway; ^d^ Mohn Medical Imaging and Visualization Centre, Haukeland University Hospital, Bergen, Norway; and ^e^ Diabetes, Endocrinology, and Obesity Branch, National Institute of Diabetes and Digestive and Kidney Diseases, Bethesda, MD, USA.

^1^R.J.B. and E.J. contributed equally to this work.

*Corresponding author

The study was performed at the University of Iceland in Reykjavik, Iceland.

Address correspondence to: Erlingur Johannsson, Professor, Centre for Sports and Health Sciences, University of Iceland, Stakkahlid, 105 Reykjavík, Iceland, [erljo@hi.is], +354-525 - 5301.

**Table S1.** Correlation matrix for response times and accuracies on the short-term working memory task.

|  |  | **1-Back**  **Response Time** | **2-Back**  **Response Time** | **3-Back Response Time** | **1-Back**  **Response Accuracy** | **2-Back**  **Response Accuracy** | **3-Back**  **Response Accuracy** |
| --- | --- | --- | --- | --- | --- | --- | --- |
|  |  | rho (*p*) | rho (*p*) | rho (*p*) | rho (*p*) | rho (*p*) | rho (*p*) |
|  | 1-Back Response Time | 1.0 (1.0) | **0.44 (< 0.001)** | **0.21 (0.01)** | -0.09 (0.3) | -0.15 (0.06) | -0.11 (0.2) |
|  | 2-Back Response Time | **0.44 (< 0.001)** | 1.0 (1.0) | **0.37** **(< 0.001**) | -0.02 (0.8) | -0.09 (0.3) | -0.08 (0.3) |
|  | 3-Back Response Time | **0.21 (0.01)** | **0.37 (< 0.001)** | 1.0 (1.0) | -0.08 (0.3) | -0.08 (0.3) | **-0.16 (0.049)** |
|  | 1-Back Response Accuracy | -0.09 (0.3) | -0.02 (0.8) | -0.08 (0.3) | 1.0 (1.0) | 0.15 (0.07) | 0.10 (0.2) |
|  | 2-Back Response Accuracy | -0.15 (0.06) | -0.09 (0.3) | -0.08 (0.3) | 0.15 (0.07) | 1.0 (1.0) | **0.19 (0.02)** |
|  | 3-Back Response Accuracy | -0.11 (0.2) | -0.08 (0.3) | **-0.16 (0.049)** | 0.10 (0.2) | **0.19 (0.02)** | 1.0 (1.0) |

Correlations were assessed using the non-parametric Spearman method. Boldface type indicates significant correlation.

**Table S2.** Correlation matrix for response times and accuracies on the visual attention task.

|  |  | **Valid Cue Response Time** | **Invalid Cue**  **Response Time** | **No Cue**  **Response Time** | **Valid Cue**  **Response Accuracy** | **Invalid Cue**  **Response Accuracy** | **No Cue**  **Response Accuracy** |
| --- | --- | --- | --- | --- | --- | --- | --- |
|  |  | rho (*p*) | rho (*p*) | rho (*p*) | rho (*p*) | rho (*p*) | rho (*p*) |
|  | Valid Cue Response Time | 1.0 (1.0) | **0.81 (< 0.001)** | **0.81 (< 0.001)** | 0.12 (0.1) | **0.18 (0.03)** | **-0.53 (< 0.001)** |
|  | Invalid Cue Response Time | **0.81 (< 0.001)** | 1.0 (1.0) | **0.75 (< 0.001)** | -0.02 (0.8) | -0.01 (0.9) | **-0.56 (< 0.001)** |
|  | No Cue Response Time | **0.81 (< 0.001)** | **0.75 (< 0.001)** | 1.0 (1.0) | 0.06 (0.5) | 0.06 (0.5) | **-0.59 (< 0.001)** |
|  | Valid Cue Response Accuracy | 0.12 (0.1) | -0.02 (0.8) | 0.06 (0.5) | 1.0 (1.0) | **0.74 (< 0.001)** | **0.25 (0.002)** |
|  | Invalid Cue Response Accuracy | **0.18 (0.03)** | -0.01 (0.9) | 0.06 (0.5) | **0.74 (< 0.001)** | 1.0 (1.0) | **0.21 (0.01)** |
|  | No Cue Response Accuracy | **-0.53 (< 0.001)** | **-0.56 (< 0.001)** | **-0.59 (< 0.001)** | **0.25 (0.002)** | **0.21 (0.01)** | 1.0 (1.0) |

Correlations were assessed using the non-parametric Spearman method. Boldface type indicates significant correlation.

**Table S3.** Working memory task performance for participants with less than seven hours of total rest time compared those with more than seven hours total rest time.

|  |  | **Total rest time ≤ 7h** | **Total rest time > 7h** | ***p*-value** |
| --- | --- | --- | --- | --- |
|  |  | **Median ± IQR** | **Median ± IQR** |  |
| ***Sleep measures of the night prior to short-term memory task*** | | | |  |
|  | n | 82 | 78 |  |
|  | 2-Back response time (ms) | 505.80 ± 130.33 | 523.44 ± 117.90 | 0.7 |
|  | 2-Back response accuracy (proportion correct) | 0.94 ± 0.06 | 0.95 ± 0.06 | 0.7 |
|  | 3-Back response time (ms) | **551.67 ± 148.06** | **514.46 ± 138.21** | **0.04** |
|  | 3-Back response accuracy (proportion correct) | 0.87 ± 0.08 | 0.86 ± 0.08 | 0.9 |
| ***Weekly sleep measures*** | |  |  |  |
|  | n | 66 | 94 |  |
|  | 2-Back response time (ms) | 517.00 ± 114.45 | 523.44 ± 131.28 | 0.8 |
|  | 2-Back response accuracy (proportion correct) | 0.95 ± 0.06 | 0.95 ± 0.06 | 0.6 |
|  | 3-Back response time (ms) | **570.32 ± 135.72** | **511.29 ± 143.71** | **0.03** |
|  | 3-Back response accuracy (proportion correct) | 0.86 ± 0.08 | 0.87 ± 0.06 | 0.2 |

All comparisons adjusted for clinical diagnosis of attention deficit hyperactivity disorder and reported weekly video game use. 2-back and 3-back response time additionally adjusted for 1-back response times. 2-back and 3-back response accuracy additionally adjusted for 1-back response accuracy. Response times and accuracies were transformed prior to analysis due to skewed distributions. IQR, inter-quartile range.

**Table S4.** Visual attention task performance for participants with less than seven hours of total rest time compared those with more than seven hours total rest time.

|  |  | **Total rest time ≤ 7h** | **Total rest time > 7h** | ***p*-value** |
| --- | --- | --- | --- | --- |
|  |  | **Median ± IQR** | **Median ± IQR** |  |
| ***Sleep measures of the night prior to short-term memory task*** | | | |  |
|  | n | 78 | 82 |  |
|  | Invalid cue response time (ms) | 356.82 ± 37.16 | 363.48 ± 44.96 | 0.3 |
|  | Invalid cue accuracy (proportion correct) | 0.91 ± 0.11 | 0.91 ± 0.09 | 0.8 |
|  | No cue response time (ms) | 383.87 ± 38.87 | 391.66 ± 45.44 | 0.7 |
|  | No cue response accuracy (proportion correct) |  | 0.96 ± 0.04 0.96 ± 0.04 | 0.5 |
| ***Weekly sleep measures*** | |  |  |  |
|  | n | 66 | 94 |  |
|  | Invalid cue response time (ms) | 358.62 ± 41.60 | 361.37 ± 39.12 | 0.99 |
|  | Invalid cue accuracy (proportion correct) | 0.89 ± 0.11 | 0.91 ± 0.07 | 0.7 |
|  | No cue response time (ms) | 387.68 ± 44.17 | 385.71 ± 48.87 | 0.8 |
|  | No cue response accuracy (proportion correct) | 0.96 ± 0.04 | 0.96 ± 0.04 | 0.4 |

All comparisons adjusted for clinical diagnosis of attention deficit hyperactivity disorder and reported weekly video game use. Invalid cue and no cue response time additionally adjusted for valid cue response times. Invalid cue and no cue response accuracy additionally adjusted for valid cue response accuracy. Response times and accuracies were transformed prior to analysis due to skewed distributions. IQR, inter-quartile range.
